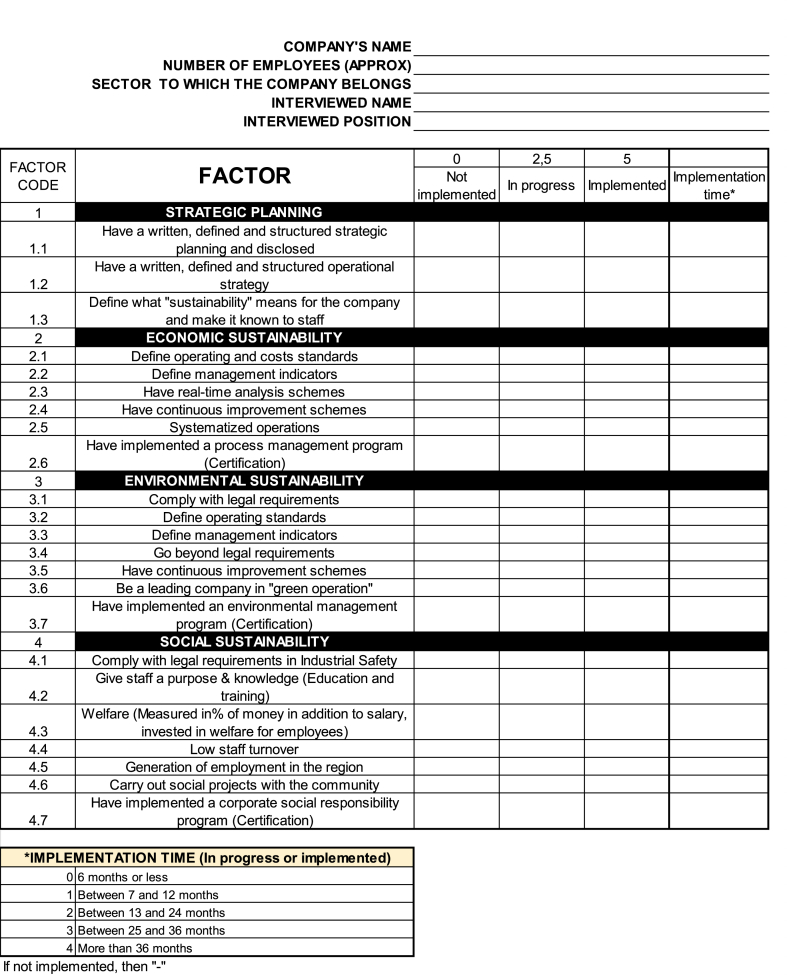# Roadmap to enhance operational excellence in emerging countries

**DOI:** 10.1016/j.heliyon.2024.e30852

**Published:** 2024-05-10

**Authors:** Rafael Henriquez-Machado, Andrés Muñoz-Villamizar, Javier Santos

**Affiliations:** a School of Economics and Management Sciences, Universidad de La Sabana, Chía, Colombia; b TECNUN Escuela de Ingenieros, Universidad de Navarra, San Sebastián, Spain

## Abstract

This paper addresses the dearth of studies examining the status of Operational Excellence (OE) implementation and offers a roadmap for enhancing OE maturity levels. The study encompassed three phases: (1) face-to-face interviews with industry OE experts in 57 companies across seven sectors, (2), classification of companies based on their maturity levels, and (3) development of a roadmap for companies to enhance their OE maturity. Through face-to-face interviews with OE experts in fifty-seven companies across various sectors, the study classified organizations into five maturity levels, with only 7 % reaching the champion level. The proposed roadmap, comprising twenty-three variables across six hierarchical levels, outlines a path for companies to progress towards champion-level OE maturity, with an estimated timeframe of 4.5–5 years from a zero level. This research contributes valuable insights into the OE implementation status in emerging countries and provides practical guidance for organizations aiming to elevate their OE maturity, emphasizing the importance of strategic planning, economic sustainability, environmental sustainability, and social sustainability variables tailored to the specific challenges and opportunities faced by businesses in emerging nations.

## 1 Introduction

Sustainability, achieved through the continuous improvement of operational processes within an organization, stands as a fundamental element for achieving business success [1]. An effective approach to attain sustainability is the implementation of an operational excellence (OE) program. In many OE programs, the focus is placed on addressing the aspects of economic, environmental, and social sustainability [2]. Various studies have traced the evolution of OE over time in diverse industries, with particular emphasis on the manufacturing sector [3]. Initially, OE primarily targeted economic sustainability [4], but subsequently, the significance of environmental and social sustainability gained traction across industries [5]. Consequently, numerous companies began adopting different methodologies to bolster their OE programs [6].

Some studies have explored OE in different organizational contexts, leading to the proposition of conceptual and maturity models [7,8]. However, there are still abundant untapped opportunities to explore the application of OE in emerging countries. The gaps between the application of OE in developed countries and emerging countries are large. Studies carried out in more than 16 developed countries indicate OE implementation level is between 80 % and 87 %, while in emerging countries it is between 34 % and 71 %. These differences are due to economic, social and cultural barriers, among others [9]. For instance, there is a dearth of recent studies documenting the maturity status of companies in emerging countries or outlining roadmaps that can facilitate their journey toward improvement. This paper endeavors to extend the maturity model developed by Henriquez et al. (2023) [10] by applying it to companies in developing countries. The aim is twofold: firstly, to assess the maturity level of companies in terms of OE and identify the constraints they face, and secondly, to develop a roadmap that enables companies in emerging countries to navigate their path toward enhancing their level of OE development.

The main objectives of this study are firstly, to assess the status of OE practices within the context of emerging countries, specifically focusing on the implementation levels of OE variables across companies from diverse sectors, complementing the study of Henriquez et al. (2023) [10]. Secondly, the research aims to identify the key factors essential for the development of a roadmap aimed at enhancing OE levels in emerging countries. Lastly, the study endeavors to craft a comprehensive roadmap delineating the trajectory towards achieving optimal standards of OE within emerging countries. This roadmap is envisioned to provide a strategic framework guiding organizations through various maturity levels, informed by expert insights and experiences gathered during the interviews conducted as part of the study.

This study aims to address two main research questions: (i) what is the status of OE practices within an emerging country context? And (ii) what are the primary factors that constitute a roadmap for improving OE levels? To answer these questions, we propose a comprehensive three-step methodology: (1) Structure and application of a face-to-face questionnaire with OE experts, (2) Classification of companies in the different maturity levels, identifying results, paradigms, and barriers, taking into consideration the size and sectors of the companies, and finally, (3) development of a roadmap towards achieving the best standards of OE in emerging countries.

The main contributions of this study are firstly, valuable insights into the current status of OE practices in emerging countries, highlighting the challenges and opportunities faced by organizations in improving their operational performance. Also, developing a detailed roadmap comprising 23 variables across 6 hierarchical levels, providing a structured path for companies in emerging countries to enhance their OE maturity and finally, practical guidance for companies aiming to elevate their OE maturity levels. Target of this study includes companies and organizations operating in emerging markets that are seeking to improve their OE performance and enhance their maturity levels.

This document is organized into the following sections: Section 2 Literature Review, which presents the conceptual framework underpinning this study; Section 3 Research Methodology, which outlines the step-by-step execution of the study; Section 4 Maturity classification, which elucidates the maturity levels of the interviewed companies. Section 5 road mapping Operational Excellence, that provides a roadmap for the advancement of companies, accounting for their limitations and paradigms; and finally, Conclusions, where the research findings are summarized, along with suggestions for future opportunities in this field of study.

## 2 Literature review

The objective of this section is to delve into the essence of OE, its connection with sustainability in industry 4.0, explore various perspectives on maturity models and roadmapping. To accomplish this, we conducted extensive research, examining literature that highlights the role of OE in promoting economic, environmental, and social sustainability within organizations. Additionally, we thoroughly reviewed studies focused on the application of maturity models in recent industry practices. Finally, an analysis of approaches to roadmapping the implementation of maturity models in relation to OE was undertaken.

### 2.1 Sustainability through OE and OE practices in industry 4.0

The concept of OE has gained significant traction in organizations in recent years [11]. One of the reasons for this is that OE enables companies to achieve sustainable growth and gain competitive advantages [12,13]. The structure of an OE program draws upon models used in Total Quality Management (TQM), Lean, Six Sigma, and Lean-Six Sigma, among others, with the aim of achieving high performance in OE [14]. A key characteristic of an OE program is its systematic approach to promoting economic, environmental, and social sustainability within organizations [9].

In the implementation of OE programs, economic sustainability holds paramount importance [15]. It serves as the foundation for business sustainability and underpins the viability of other sustainability dimensions [4,15,16]. To attain economic sustainability, OE relies on the establishment of operational standards that ensure process and product compliance, as well as the use of management indicators to measure goal attainment and continuous improvement initiatives [17].

Likewise, OE also addresses environmental sustainability and social sustainability [[18], [19], [20], [21]]. Environmental sustainability goes beyond mere adherence to legal requirements and aims to align operational processes with environmental stewardship. This entails the definition of operating standards, management indicators, and continuous improvement schemes [22]. Maturity models have been developed to guide organizations from a beginner's level of environmental compliance to becoming recognized as champion companies [23].

Social sustainability, plays a vital role within OE, acting as a pillar for achieving organizational objectives. It enables businesses to cultivate a long-term organizational culture that supports continuous improvement efforts [24]. Organizations pursuing their objectives recognize that ensuring the well-being of their employees is crucial, as they are instrumental in achieving goals [20]. Key aspects considered in social sustainability include purpose, knowledge, and welfare according to Henriquez et al. (2023) [10]. Firstly, the purpose allows the worker to understand what their role is, the reason for it and its alignment with the strategic objectives. Secondly, knowledge gives the employee the "know how" they will need to fulfill their role: training activities, training, etc. Finally, the welfare that allows the worker to feel part of the team and committed to the organization [21]. Some of the aspects of welfare include fair salaries, clean and tidy workplaces, complete and well-maintained tools, after-work activities, and worker happiness, among others [25].

### 2.2 Maturity models in OE

Maturity models are frequently utilized tools for assessing a company's readiness to improve its operations [26]. Numerous studies focus on maturity models in terms of economic, social, and environmental sustainability. Additionally, maturity models are applied to methodologies such as Total Quality Management (TQM) [27], Lean [22], Six Sigma [26,28], and Industry 4.0 [26,29,30], which form the foundation of contemporary OE concepts. For instance, there are maturity models that outline different stages for implementing Industry 4.0 tools, based on identifying operational scenarios [31]. Other studies delve into Industry 4.0 micro-foundations through case studies [29], explore the maturity of technological tools in the manufacturing industry [30] and even develop models for IT management [32]. OE includes the implementation of management indicators as measurement tools for standard compliance [7], and thus there are studies on maturity models for implementing indicators in operational processes [33,34].

From an environmental perspective, which is another crucial aspect of OE [35], various maturity studies have been conducted. Examples include: exploring the structure of sustainable operational processes [27], developing environmentally friendly products based on clean processes [36] and implementing a comprehensive maturity scheme focused on environmentally sustainable practices [23]. Some studies integrate maturity models that encompass economic aspects, management indicator schemes, technological implementation, and environmental considerations [22,37]. These studies assess the impact of implementing Lean or Six Sigma tools across different operational aspects [28,37].

There are studies on maturity models in social sustainability [38]. For instance, a study conducted in the public sector employed a maturity model to measure operational processes and the path toward social sustainability [39]. Other studies examine the relationship between maturity, organizational culture, work environment, and economic outcomes [40]. Some studies even incorporate the use of Lean tools based on worker maturity to establish sustainable operational standards and continuous improvement schemes [29].

Considering various maturity models closely related to OE, a study specifically focused on all aspects of OE implementation in emerging countries [10]. In the study, 23 OE variables were validated through interviews with general managers, operations managers, and industry experts within 49 companies across different sectors. The findings led to the determination of five maturity levels: basic level, beginner level, training level, innovative level, and champion level. The 23 variables were organized within these maturity levels, as shown in Table 1, that defines the factors that constitute an OE program, including the strategic factor (strategy) which emphasizes the importance of having a well-defined, clear, and communicated strategic planning with employees [25,41]. Additionally, economic sustainability (Ec. S.), environmental sustainability (En. S.), and social sustainability (S.S.) are integral components of an OE program (see Table 2).

**Table 1:** Relation variables, maturity level and factors in an OE study [10].

Variable number	OE Variables	Maturity level	OE Factors
1	Strategic planning & disclosed	Basic	Strategy
2	Operational Strategy	Beginner	
3	Definition of sustainability	Basic	
4	Operating & costs standards	Basic	Ec.S.
5	Indicators	Basic	
6	Real-time analysis	Champion	
7	Continuous improvement	Beginner	
8	Systematized operations	Champion	
9	Certification	Training	
10	Legal requirements	Basic	En.S.
11	Operating standards	Beginner	
12	Indicators	Training	
13	Beyond legal requirements	Champion	
14	Continuous improvement	Beginner	
15	Green leader	Champion	
16	Certification	Innovative	
17	Legal requirements	Basic	S.S.
18	Purpose & knowledge	Basic	
19	Welfare	Innovative	
20	Low staff turnover	Training	
21	Employment	Training	
22	Social projects	Innovative	
23	Certification	Training

**Table 2:** Number of companies interviewed by size and sectors.

Size	Small	Medium	Large	Total
Sector				
Food & drinks	6	6	2	14
Services	6	4	4	14
Manufacture	4	1	4	9
Construction	3	3	0	6
Oil/gas	0	4	1	5
Transport & logistics	3	2	0	5
Agriculture	3	0	1	4
Total	25	20	12	57

### 2.3 Roadmapping in OE

Battistoni et al. (2023) [42] highlighted recent advancements in roadmapping related to OE. Roadmaps are valuable tools for organizations as they provide guidance for progressing from one level to another in pursuit of specific objectives, such as OE [43]. Various methodologies are employed in developing roadmaps, including SWOT analysis (strengths, weaknesses, opportunities, and threats), brainstorming, business model canvas, balanced scorecard, and cost-benefit analysis, among others [44].

Within the structure of a roadmap, the steps that follow organizations from a basic or initial state to a state of full implementation must be defined [45]. The steps may involve formulating a transition strategy, selecting activities for improvement within a project, defining the scope of the improvement, choosing the appropriate technologies and techniques to initiate the project, evaluating the achievements of the project, and launching subsequent initiatives. This iterative process continues until companies achieve the desired level of maturity based on the implemented measures [46,47].

Some authors have employed interviews to develop roadmaps, where the variables to be fulfilled are defined based on a maturity model [48]. Through expert interviews, input is gathered on the time required to implement the variables and their prerequisites. By averaging these times, it becomes possible to structure the implementation period and identify the sequential activities and variables to be implemented [49]. Many studies on OE program implementation follow a Project Portfolio Management (PPM) approach [50]. PPM is a continuous process that focuses on project-oriented initiatives aimed at delivering business value, with profitability as the primary objective. The problems addressed in PPM are tackled using methods such as comparative analysis, scoring, optimization, or simulation [51].

In Fernandes et al.'s (2023) [52] study, a strategic roadmap was developed to highlight the transformative potential of additive manufacturing (AM) in production processes, focusing on technologies like Fused Deposition Modeling (FDM), Stereolithography (SLA), and Selective Laser Sintering (SLS). Factors influencing AM adoption, including economic, labor shortages, and regulatory considerations, were examined. Anticipated shifts in business models towards increased home-based printing and consumer involvement were emphasized. The study also explored opportunities in aerospace, automotive, and healthcare sectors catalyzed by AM integration, particularly within the context of Portugal. A methodological triad involving planning, formulation, and iterative review stages was employed, utilizing various research methodologies such as literature synthesis, questionnaire analysis, expert consultations, and database utilization. This comprehensive approach aimed to provide Portuguese enterprises with a strategic tool for effective AM integration and innovation exploration.

## 3 Research methodology

**Fig. 1:**
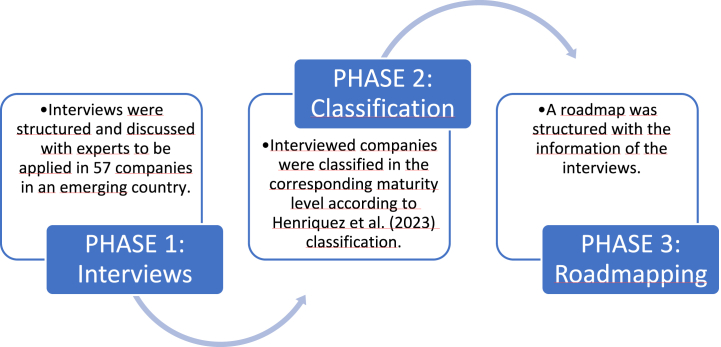
Structure of the research method.

The methodology employed in this study is grounded in exploratory research, employing a mixed methods approach. This multifaceted strategy systematically addresses both qualitative and quantitative dimensions in a sequential manner, as expounded by Ref. [53]. The overarching objective of this research endeavor was to conduct a rigorous empirical assessment, quantifying the maturity of OE across various entities within an emerging contextual framework. To execute this objective meticulously, the research was compartmentalized into three distinct phases, each meticulously outlined in Fig. 1.

### 3.1 Interviews

For the formulation of the interview instrument, we drew upon the maturity levels and variables elucidated in the study conducted by Henriquez et al. (2023) [10] and described in Table 1. This pioneering study identified a comprehensive set of twenty-three variables constituting a holistic OE program tailored specifically to emerging countries. Each of these variables resides within one of four overarching facets delineating organizational sustainability through operations: Strategic planning, economic sustainability, environmental sustainability, and social sustainability. The study was adapted and applied to the specific context of emerging nations through a structured approach [10]. The model is tailored to suit the needs of businesses in emerging countries.

The study first identifies the key factors essential for operational excellence in the context of Industry 4.0 and emerging countries. These factors are derived from a thorough literature review and are specific to the challenges and opportunities faced by businesses in emerging nations. The variables identified through the literature review are organized into five common factors: strategic planning, economic sustainability, environmental sustainability, social sustainability, and certifications/credentials. These factors encompass a range of topics relevant to operational excellence in emerging countries. A structured questionnaire is developed to capture the specific needs and challenges of businesses in emerging countries. This questionnaire is designed to assess the maturity levels of organizations in terms of operational excellence and guide them towards improvement.

The maturity model defines four or five maturity levels that businesses can progress through to enhance their operational excellence. Each level represents a stage of development and improvement, with recommended activities and indicators to guide organizations in their journey towards excellence. The variables and maturity levels proposed in the model are validated through expert validation, ensuring that they are relevant and applicable to the specific context of emerging nations. This validation process helps to refine and enhance the model for effective implementation. By considering the unique economic, political, and social conditions of emerging countries, the maturity model provides a framework for organizations to assess their current status, identify areas for improvement, and implement strategies to enhance their operational excellence in alignment with Industry 4.0 principles.

The interview for this study was structured based on the twenty-three variables and to achieve the objectives of this study, a three-step method was followed to be able to apply it [54,55]. In the initial phase, we expounded upon the concept of OE and presented the overarching objective of our study. In the subsequent step, we provided a comprehensive description of the twenty-three variables, seeking to ascertain the extent to which each variable was implemented within the organization where questionnaire was administered. Finally, the third step delved into probing the motivations behind an organization's decision to either conform to or deviate from these variables. This last step facilitated an understanding of whether these implementations stemmed from internal initiatives, directives from parent companies, or a response to competitive or industry-wide trends. Moreover, it offered insights into the barriers and paradigms inhibiting the integration of these variables within each organization [54].

To assess the status of each variable, we employed a qualitative Likert scale. A Likert scale is a measurement tool used in social and psychological research to assess participants' attitudes, opinions, or perceptions. It consists of a series of statements or items on a specific topic, along with a range of response options, from "strongly disagree" to "strongly agree." Participants indicate their level of agreement or disagreement by selecting an option that best reflects their opinion. This scale provides quantitative data that can be statistically analyzed to understand individuals' attitudes and perceptions regarding the study topic [56]. It is important to mention that the variables used according to Henriquez et al. (2023) [10] were defined by experts in industry.

The Likert scale used in this study comprised of three distinct categories: "Not implemented," "In progress," and "Implemented," thereby delineating the degree of implementation [57]. After structuring the interview, it was subjected to a rigorous two-tier review process. Initially, it underwent scrutiny by five academics, each boasting over 15 years of experience, drawn from a prestigious academic institution. Following this, validation from five industry experts was sought to ensure the clarity and relevance of the interview questionnaire. The detailed interview structure can be accessed in Appendix A.

In total, invitations were extended to one hundred and sixty companies located in Bogotá and the central savannah region of the Colombian capital to participate in our research. Ultimately, we conducted face-to-face interviews with fifty-seven companies that consented to partake in the study. These organizations spanned across seven diverse industry sectors and were stratified by size, with 44 % categorized as small, 35 % as medium, and 21 % as large enterprises. The interchanges with each company ranged from one to one and a half hours. The principal respondents for interviews were primarily operations managers, aligning with the primary target. However, in select cases, it was engaged with general managers or senior operational positions, including manufacturing managers, factory managers, or logistics managers. It is noteworthy that all interviewees held managerial roles within their respective organizations.

Before conducting the in-person interviews with all the participants, a preparatory letter was sent to each interviewee. This introductory communication discussed the interview's purpose, its origin, and underscored the empirical nature of the study. It aimed to provide interviewees with a preliminary understanding of the impending engagement. However, during the actual meeting, a comprehensive explanation was presented, expounding upon the concept of OE and delving deeper into the content of the introductory letter. The essence of this exercise was to glean the most precise and insightful information possible from operational leaders within the participating companies. As we traversed the various facets of OE, including strategic planning, economic sustainability, environmental sustainability, and social sustainability, interviewees were meticulously briefed about each of these dimensions to ensure a shared understanding.

In the course of addressing each of the twenty-three variables and collecting responses from interviewees, they were embarked on a further exploration of the implementation process. Specifically, for variables marked as "implemented," additional insights into the nuances of the implementation journey was sought. Interviewers inquired about the motivations underpinning the decision to implement a particular variable and sought an approximation of the timeline involved in reaching this stage. For those variables categorized as "in progress," the exploration delved into the progress made thus far. It was probed into the motivations that catalyzed the initiation of these implementations and the anticipated timeline for completion. In cases where interviewees indicated that a variable was "not implemented," they were embarked on a comprehensive exploration of the rationale behind this decision. Interviewers inquired whether there was a desire to implement the variable and, if so, what were the obstacles or paradigms hindering its adoption.

By adopting this meticulous and holistic approach during the interviews, we aimed to capture a comprehensive and nuanced understanding of each organization's OE journey, while also illuminating the motivations and challenges that shaped their decisions regarding the integration of these vital variables.

### 3.2 Classification

Following the acquisition of survey results, a meticulous classification process was employed to categorize the studied companies into one of five distinct maturity levels: Basic, Beginner, Training, Innovative, and Champion. This classification criteria aimed to reflect the varying degrees of implementation and integration of the twenty-three analyzed variables.

The highest echelon of maturity, the Champion level, was reserved for companies that had successfully implemented all twenty-three variables, signifying a comprehensive embrace of OE practices. Companies achieving the Innovative level demonstrated substantial progress by implementing variables that encompassed Basic, Beginner, Training, and Innovative stages. This denoted a commendable commitment to enhancing organizational performance through a multifaceted approach. The Training level was ascribed to organizations that had diligently incorporated variables spanning Basic, Beginner, and Training stages. This demonstrated an effort to cultivate operational excellence. Companies designated as Beginner had taken the crucial first steps by implementing Basic and Beginner variables, indicating an initial foray into the realm of OE. Lastly, entities falling into the Basic level had yet to integrate the variables characteristic of Beginner, Training, Innovative, or Champion stages. This classification highlighted a foundational stage in their OE journey.

To visually distill and comprehend the levels of implementation of each variable across the surveyed companies, a heatmap analysis was employed. The heatmap, selected for its capacity to offer an intuitive visual representation of data, facilitated the identification of commonalities and disparities among organizations. This enabled the discernment of which variables were consistently met across companies and which were less frequently addressed [58]. The intent was to discern patterns, both in terms of similarities and differences, for each maturity level.

Lastly, utilizing the wealth of information gleaned from expert insights during the interviews, encompassing company initiatives, motivations, paradigms, and restrictions, a comprehensive roadmap was meticulously constructed. This roadmap would serve as a strategic guide, synthesizing the collective wisdom and experiences of the interviewed organizations to facilitate future OE endeavors.

### 3.3 Roadmapping

The development of the OE roadmap in the study closely adheres to a methodological approach inspired by Lu and Weng (2018) [49]. The proposed architecture for the roadmap is defined in two ways [42]: firstly, it establishes a sequential path for each organization to ascend to higher levels of OE maturity. This necessitates an understanding of the variables, their significance, and their corresponding maturity levels. Secondly, it endeavors to determine the timeframes required for an organization to implement each of these variables. This comprehensive approach enables the delineation of a step-by-step progression and the estimation of the total time it may take for an organization to attain the highest level of OE implementation.

During the interviews, operational leaders were queried regarding the temporal aspects of OE variable implementation. Specifically, they were asked to provide insights into the time taken for variables already implemented and, for those in progress, an estimate of the expected completion timeline. This invaluable data was aggregated for each variable and used to measure the anticipated time required for an organization to implement each OE variable.

Furthermore, this dataset was instrumental in calculating the overall average time needed for an OE program to transition from a Beginner level to the coveted Champion level. Importantly, the maturity level of each variable, as per the classification by Henriquez et al. (2023) [10], was also considered. This allowed for an understanding of the prerequisites and dependencies associated with each variable, further enhancing the precision of the roadmap. By adopting this systematic and data-driven approach, this study aims to provide organizations with a well-informed and strategic roadmap for navigating the intricate terrain of OE, accounting for both the sequence of variable implementation and the temporal dimensions required for each stage of progress.

4. MATURITY CLASSIFICATION

**Fig. 2:**
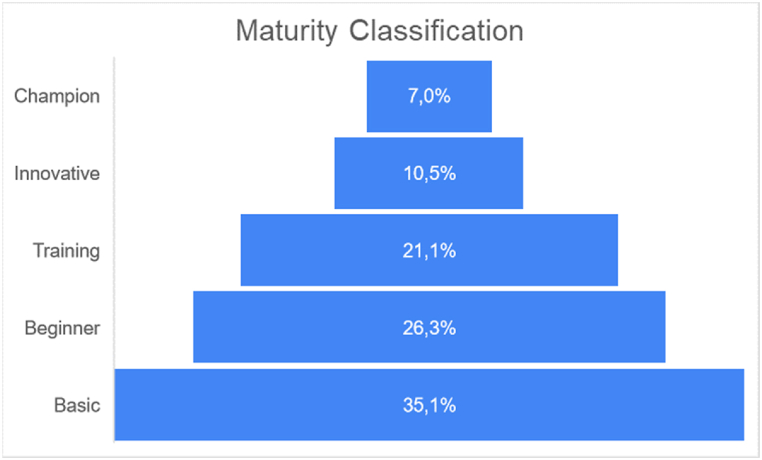
Maturity classification of the studied companies.

The analysis of interview results has led to a noteworthy classification of companies based on their maturity levels, as illustrated in Fig. 2. A significant finding emerges from this classification, with a substantial majority of companies, amounting to 82.5 %, positioned at the Training level or below. Specifically, 35 % of the organizations fall within the Basic level, while a mere 7 % have achieved the prestigious Champion level.

The results also show that all champion level companies are large-size and correspond to 33 % of companies of that size. Likewise, there are none of large-size companies at the basic level. An opposite effect is observed in small-size companies. Where none of them that are at the champion or innovative levels. Only 12 % of these companies appear as the highest level in training, while the vast majority are at the basic level, corresponding to sixteen companies or 64 % of the small-size. As for medium-size companies, they are distributed from the basic to the innovative level in a relatively similar way. At the beginner level there is the largest participation, with seven companies that correspond to 35 % of the total in this size. Table 3 summarizes the OE maturity level by size.

**Table 3:** Results of OE maturity classification per size.

Size Level	Small	Medium	Large	Total
Champion	0	0	4	4
Innovative	0	4	2	6
Training	3	5	4	12
Beginner	6	7	2	15
Basic	16	4	0	20
Total	25	20	12	57

**Fig. 3:**
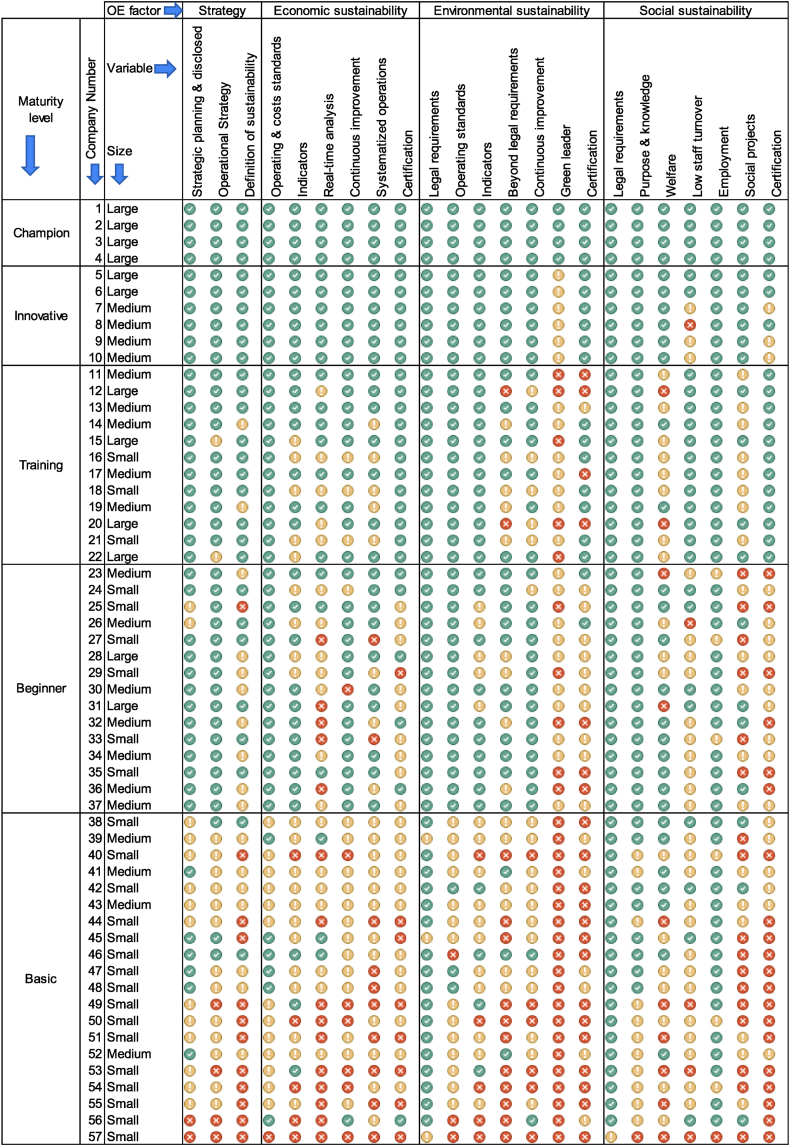
Heat map chart of the implementation of each OE variables per company.

The analysis of the fifty-seven companies based on each of the twenty-three variables has revealed intriguing patterns and differences across various maturity levels, as depicted in Fig. 3. This heatmap provides a visual representation of compliance with each variable, denoted in green for full compliance, yellow for variables in the process of implementation, and red for variables that are not implemented. The heatmap is organized in descending order based on the implementation of variables and the maturity level of the companies.

The first four companies (number 1 to 4) in Fig. 3, that are classified at the champion level meet all the variables defined for an OE program. Another feature is that all four are large-size. The next six companies (number 5 to 10) do not qualify as Champion and are at the innovative level. It is observed that there are no more than three variables that have not yet met or are in the process of becoming Champion. All of them are characterized because they have been working on being green leader companies and number 5 and number 6 only have that pending variable to be Champion. It can be said that they are close to being. For their part, companies' number 7 to 10, apart from working on the green leader's variable, have one or more other variables that are in process or have not been implemented. These last four companies agree that the variables of low staff turnover and certifications are the ones that are pending. In the case of company number 8, although it has certifications, it currently does not measure and is not interested in measuring its staff turnover. In the interview, the operation manager of company number 8 stated that they do not need to measure it since they offer what they consider to be an excellent work environment, so the employees who value it stay, and those who do not look elsewhere for what they have not found there. In this group of six companies, there are already four medium-sized accompanying two large-sized.

The companies that are at the Training level (from 11 to 22), are characterized by meeting or at least being in the process of implementing the variables of the strategy and economic sustainability factors. None of the twelve companies that are at that level have red colors in the variables of the first two factors. The typical characteristics observed in these companies are that they all have strategic planning implemented (variable 1), they have defined operating and cost standards in their processes, and they have process management system certification. Regarding the variables of the environmental sustainability factor, the general behavior is that apart from compliance with legal requirements, they have defined environmental operating standards and metrics to measure it. The environmental indicators in this factor seem to be more consolidated than even those in the economic sustainability factor. As for the social sustainability factor, except for the welfare and social projects variables that mostly do not have them in green color, they show significant progress in general.

At the beginner's level, delineated by companies ranked from 23 to 37, the majority, with the exception of two entities, exhibit a proactive approach towards strategic planning and possess a well-defined operations strategy. In economic sustainability, unlike the training level, most of them no longer have a labor certification under standardized processes. Only five of the fifteen at this level have it. At this level, the lack of indicators in the environmental sustainability factor begins to be seen in some companies, although environmental operating standards and continuous improvement schemes are a trend at this level. Low staff turnover and employment in the region are not measured within the social sustainability variables.

Finally, at the Basic level is where there is a lack of OE practices. It was observed how the companies hardly comply with legal requirements and make little progress in strategic factors. Companies like number 39, number 45 and number 57 still have legal variables that are in progress. In the case of the first, which is medium-sized, they stated in the interview that they believed they were complying with environmental legal requirements, however, a few days prior to the interview of this study, they received a fine from the local environmental authorities, due to some discharges out of specifications. For this reason, in the interview they marked the point as in process. Company number 45 manifests some problems with its emissions but has been working on the implementation of a bag filter to be within the respective specifications. Company number 57 is a small-sized company with less than six months of experience at the time of the interview and is in the structuring process.

## 4 Roadmapping operational excellence

The study developed by Henriquez et al. (2023) [10] can significantly contribute to the structure of an operational OE, particularly in the context of emerging countries. There were identified 23 variables crucial for shaping an OE roadmap, particularly pertinent in emerging countries. **Strategic planning variables** facilitate alignment of operational goals with overall business strategy, emphasizing corporate strategy alignment, communication, and operational strategy development. **Economic sustainability variables**, including efficiencies and continuous improvement, enable optimization of operations for cost-effectiveness and profitability within resource-constrained environments common in emerging countries**. Environmental sustainability variables** promote eco-friendly practices by emphasizing compliance with regulations, green leadership, and continuous improvement, addressing growing environmental concerns in these regions. **Social sustainability variables** prioritize compliance with safety standards, social responsibility practices, and legal requirements to foster positive societal impact, enhancing reputation and stakeholder trust.

Moreover, certifications and credentials related to process, environmental, and social standards signify OE achievements and bolster credibility, competitiveness, and market positioning for businesses in emerging countries. In this context, the proposed OE roadmap structured around these variables provides a comprehensive framework for assessing, planning, and implementing initiatives tailored to the unique challenges and opportunities faced by organizations in emerging markets. By addressing strategic planning, sustainability, and certifications, businesses can navigate their operational improvement journey effectively, contributing to sustainable success amidst the dynamic business landscape of emerging countries. Considering the 23 variables, and the results of the face-to-face interviews, the roadmap of OE for emerging countries can be developed.

On the interviews, experts in organizations were asked about the time it took them to implement the OE variables that they already have. In cases where the variable was in progress, they were asked how long they estimated it would take to be implemented. For the variables not implemented, an estimated time was not asked. Table 4 summarizes the results by company and factor using a Likert scale. Zero (0) represents an implementation time of 0,5 years or less. One (1) represents between 0.5 and 1 year. Two (2) means between 1 year and 2 years. Three (3) is between 2 years and 3 years. Finally, four (4) refers to a time greater than 3 years.

**Table 4:**
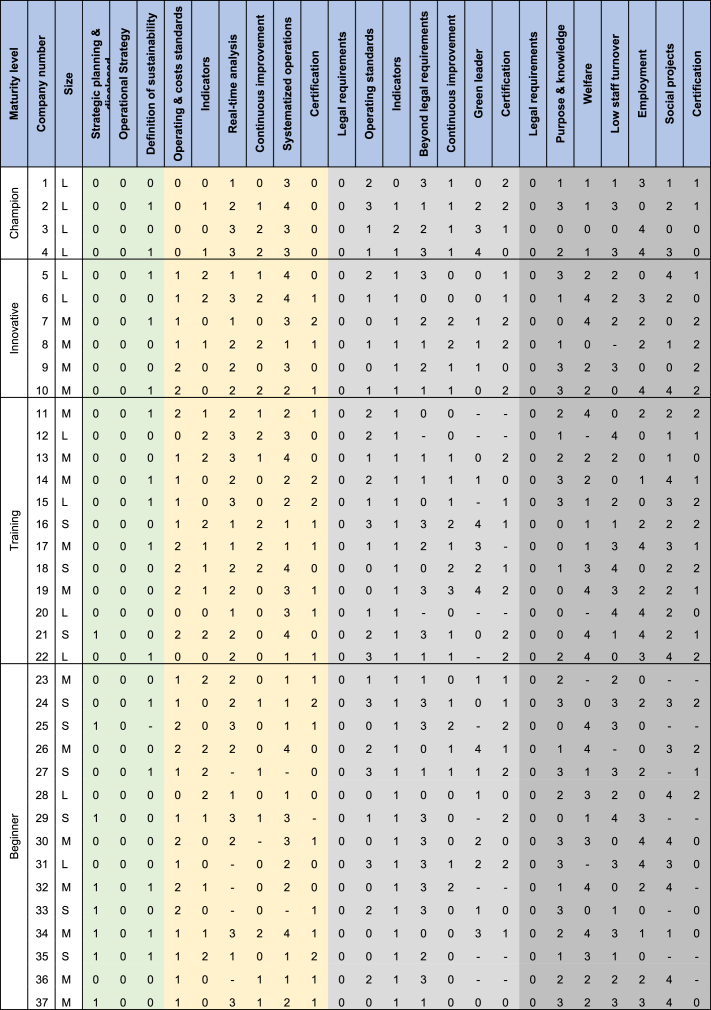

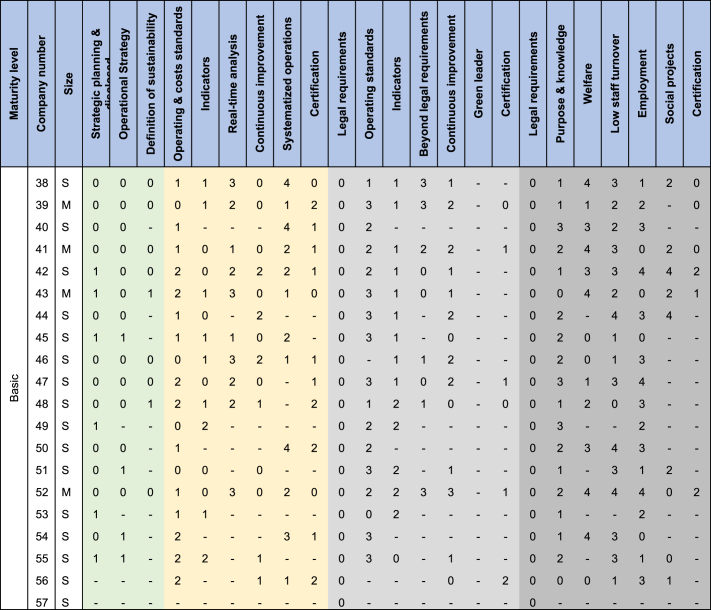
Time that each company spent for each OE variable implemented or estimated to spend if the OE variable is in progress. (In size L = Large, M = Medium, S= Small).

Note: Operational Excellence factors in Table 4 are colored as Strategy, economic sustainability, environmental sustainability, and social sustainability.

Once Table 4 was made, following the Peng & Lu (2018) [49] methodology, the average time that companies spent for each variable was calculated. In this way, it would be known how long an organization can take to implement a specific OE variable, on average. Subsequently, the variables were organized according to their prerequisites, defined in the study by Henriquez et al. (2023) [10]. For example, in first instance to talk about OE, companies must comply with legal requirements and establish strategic planning. Second, define the operations strategy and what the company defines as sustainability. Without an operations strategy, it is difficult to advance in OE, as it is unknown which activities are going to add value or not. Third, define operating standards and metrics to measure its management (both economic and environmental). Simultaneously, variables such as Purpore & knowledge, welfare and employment can be implemented.

Fourth, work begins on continuous improvement schemes (Economic and environmental). For those processes, it is important to have defined operating standards, otherwise it is unknown what is going to be improved. In parallel, it is possible to begin to implement real time analysis and systematized operations in economic sustainability because it depends on operating standards and management indicators. The same happens with the Beyond legal requirements variable in environmental sustainability, as to go beyond the legal requirements, is necessary, on the one hand, to comply with them and, on the other hand, the management indicators that demonstrate it. Likewise, in social sustainability, Low staff turnover can be implemented, considering that it depends on people having purpose & knowledge. Social projects that depend on welfare, can also begin to advance in this fourth place. Social projects depend on Welfare because internal social sustainability is ensured first and then external [22]. Fifth, the certification variables can be implemented in all the sustainabilities. To be certified, it is necessary to have defined continuous improvement schemes. Finally, from environmental sustainability, once all other variables are met, it is possible to start working on being a Green leader.

**Fig. 4:**
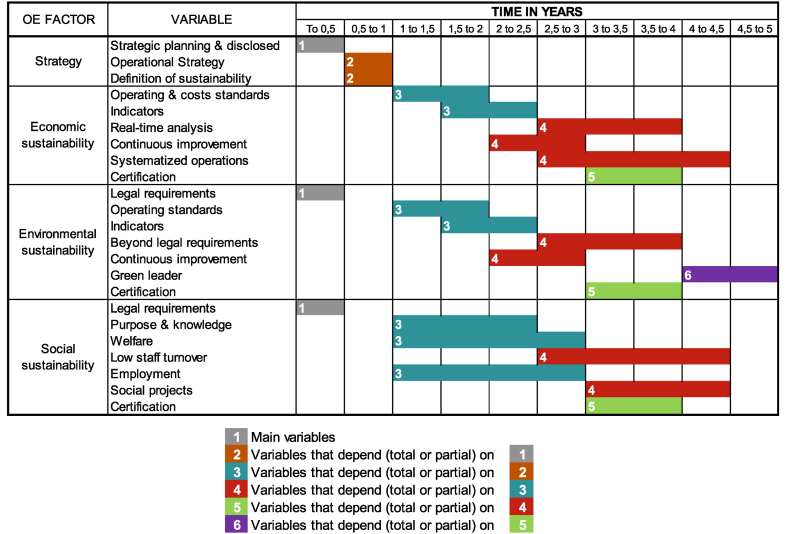
Average time in years to implement each OE variable considering the hierarchy defined by Henriquez et al. (2023) [10].

**Fig. 5:**
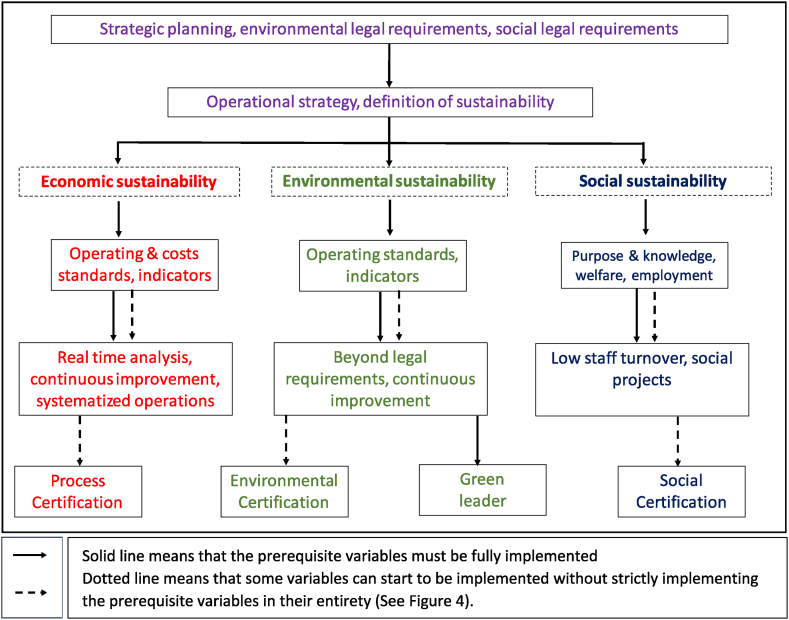
Roadmap development for OE.

Once the results of Table 4 and the hierarchy mentioned above are ready, Fig. 4 is developed and there it was estimated that it can take an average of 4.5 years to reach the champion level. Similarly, Fig. 5 illustrates the hierarchy defined to gradually implement the different variables of OE and represents the roadmap development for operational excellence.

**Fig. 6:**
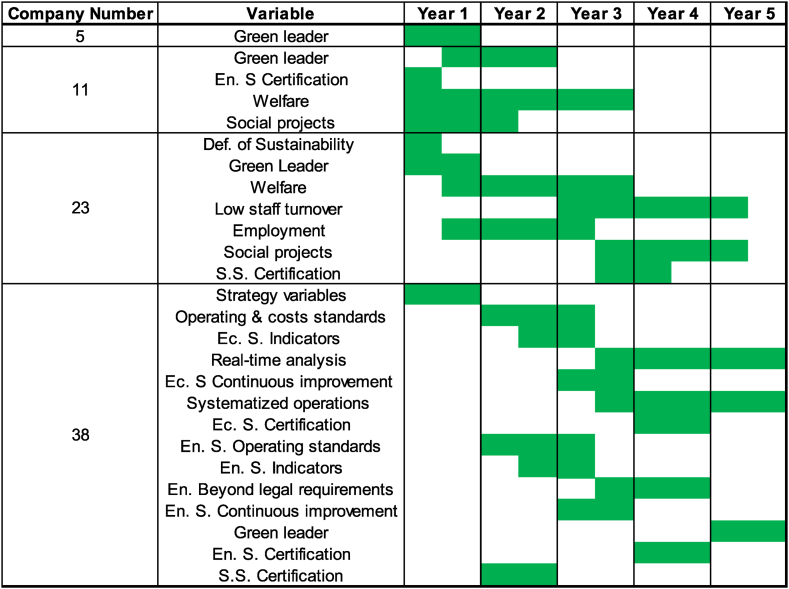
Expected time randomly selected companies can take to achieve Champion Level.

Additionally, the designed roadmap was used in this text in a company from each level below the Champion. It was randomly decided to select the first company from each of the levels. In the case of the Innovative level, company number 5 was chosen, for the Training level, number 11, for the Beginner level, number 23, and for the Basic level, company 38. The results are depicted in Fig. 6.

Assuming that companies would like to reach the Champion level, have the intention, and possess the necessary resources to achieve Champion level, the following can be observed for each company.

• Company number 5: It only needs to become a Green Leader, for which an approximate period of one year is estimated, considering the average times from Table 4 and the progress that Company 5 already has with the variable. As seen in Fig. 3, implementation is already underway.
• Company number 11: It is currently engaged in Welfare and Social Projects. Considering its initiation of the certification process and subsequent transformation into a Green Leader, it may take approximately 3 years.
• Company number 23: It is working on sustainability definition, aiming to become a Green Leader, focusing on Low Staff Turnover and Employment. It still needs to begin work on Welfare, Social Projects, and S.S. Certification. It is estimated that this company may take around 4.5 years, primarily because Welfare alone would take over 2 years, contingent on sustainability definition. Upon completing Welfare implementation, work can commence on Social Projects and certification.
• Company number 38: This company has the particularity of redefining its corporate strategy. While it currently has a defined operations strategy and sustainability definition, a change in the company's strategy may imply alterations in these last two variables. Anticipating potential changes in operations strategy and sustainability definition, we allocate one year for this analysis. The company states it is working on all six variables of the economic sustainability factor. Regarding environmental sustainability, it is only complying with legal requirements. Four of the remaining six variables are in the implementation phase, and two have not yet been initiated. The social sustainability factor variables are implemented, except for certification, which is in progress. Under this scenario, it is estimated that the company may take 5 years to reach the Champion level.

## 5 Discussion

The assessment of OE practices within the context of an emerging nation reveals that merely seven percent (7 %) of companies have achieved champion status, whereas thirty-five percent (35 %) find themselves at the basic level. The basic tier commands the largest share of companies within the study cohort. Furthermore, all champion-level enterprises are large-size, with no entity of such stature relegated to the basic level. Local large-size companies exhibit remarkable organizational structures, predominantly engaged in exporting their products while upholding high process standards. Innovative companies, conversely, are striving to ascend to the champion level. In essence, their endeavors revolve around various key variables, including their pursuit of green leadership, reduction of staff turnover, and the attainment of certifications that validate their social practices.

Intriguingly, companies at the innovative level may fall within the purview of either large-size or medium-sized entities. While entities at the Trainer and Beginner levels, many prerequisites must be met before an ascent to a higher OE tier. These companies uniformly emphasize their adherence to well-defined operational standards, a relentless commitment to continuous improvement, performance metrics, and unwavering compliance with legal requisites. Remarkably, among companies relegated to the basic level, many are smaller organizations who at least meet the baseline of legal compliance. This phenomenon can be attributed to the transitional shift from informal to formal corporate structures, a challenge undertaken by diminutive enterprises that strive tenaciously for survival amid adversity.

In a broader context, the interviews conducted revealed a shared commitment among companies to proactively enhance their OE standing. It is abundantly evident that some companies face greater challenges in their quest for advancement, primarily due to their constrained economic circumstances. Paradoxically, paradigms such as the perceived need for substantial investments, the limited value attributed by clients to OE scaling, and the anticipated competitive challenges associated with achieving champion level are called into question.

A meticulously structured roadmap was devised to aid organizations in their ascent through the OE ranks, focused upon the implementation of twenty-three distinct variables, each requiring a carefully orchestrated sequence. This hierarchical roadmap imposes structural coherence as companies transition from a basic to a champion level. Additionally, bearing in mind the average timeframes requisite for organizations to adopt these diverse OE variables, a temporal framework has been proposed to facilitate the transition from one tier to another. In essence, it is estimated that, assuming unimpeded continuous efforts and unwavering support from senior management, a company may require approximately 4.5 years to ascend to the champion level. It is noteworthy that the phases associated with the lengthiest implementation periods, encompassing 2 years feature the systematization of operations, welfare, low staff turnover, and employment.

Important paradigms have surfaced within companies concerning the implementation of specific variables and the associated tier advancement. In some cases, the client's perception of value is pivotal, particularly when related to internal social aspects such as seeking low staff turnover or working based on their welfare. Some entities exhibit limited interest in assuming leadership roles in environmental sustainability or venturing beyond legal requisites. It is important to acknowledge that these dynamics may be rooted in the unique cultural conditions of the country and warrant consideration in future research endeavors.

Addressing challenges associated with certain variables in the context of OE in emerging countries can significantly enhance organizational value. Factors such as the complexity of implementation, resource limitations, regulatory compliance, cultural and organizational resistance, scalability, and adaptability play crucial roles in determining the success of OE initiatives. For instance, the complexity of implementing advanced practices or technologies may pose challenges for organizations with lower maturity levels or limited resources, necessitating innovative solutions and strategic resource allocation to enhance competitiveness.

Furthermore, regulatory compliance, especially in areas such as environmental sustainability and social responsibility, presents challenges in emerging countries with evolving regulatory frameworks. Overcoming these challenges not only mitigates risks but also enhances reputation and contributes to sustainable development. Cultural and organizational resistance may impede the adoption of certain variables, emphasizing the importance of fostering a culture of continuous improvement and employee engagement to drive organizational change.

Additionally, addressing challenges related to scalability and adaptability ensures that OE practices remain effective across different organizational sizes and contexts. Successfully overcoming these challenges provides organizations with a competitive advantage by demonstrating agility, innovation, and resilience in navigating the dynamic business landscape of emerging countries. In conclusion, addressing challenges associated with specific variables in OE initiatives adds significant organizational value by optimizing processes, ensuring compliance, fostering cultural change, promoting scalability, and driving competitive advantage.

## 6 Conclusions and future research

The study yielded several significant quantifiable findings. Firstly, it classified companies into five distinct maturity levels: Basic, Beginner, Training, Innovative, and Champion. This classification aimed to delineate the varying degrees of implementation and integration of the twenty-three analyzed variables within different organizations. Additionally, the study utilized a qualitative Likert scale to assess the implementation status of each variable, categorizing them as "Not implemented," "In progress," and "Implemented." This approach provided a quantitative measure of the degree of implementation across diverse organizations, offering insights into the overall progress of OE initiatives.

Furthermore, the study conducted face-to-face interviews with fifty-seven companies from various industry sectors in Bogotá and the central savannah region of the Colombian capital. These organizations were stratified by size, with 44 % classified as small, 35 % as medium, and 21 % as large enterprises. The distribution of participants across different sectors and sizes provided valuable insights into the representation of industries within the study sample. Finally, the study likely calculated average scores for different maturity levels based on the responses gathered during the interviews. These scores elucidate the overall progress and performance of companies at each maturity level, offering a comprehensive understanding of the OE landscape in emerging countries.

This study makes a substantive and practical contribution to both industrial and academic realms, harboring the potential for economic and commercial ramifications in emerging nations. The study provides companies with a strategic planning tool by utilizing this roadmap, planning, and executing initiatives aimed at enhancing their OE maturity. In addition, companies can utilize the insights from the study to drive performance improvement initiatives by assessing their current standing in terms of OE. Also, this study facilitates the integration of sustainability practices into OE initiatives incorporating economic, environmental, and social sustainability considerations into their operational strategies. Academically, this work provides a foundational toolset for illustrating the OE program's roadmap. Moreover, the study has the capacity to foster community improvement through incentivizing companies to elevate their OE maturity, thereby generating positive external social impacts and promoting national development.

As an inherent limitation, it is crucial to acknowledge that the findings of this study draw upon the insights of industry experts who are well-versed in OE concepts and objectives, each possessing unique backgrounds and experiences that may have influenced results, potentially introducing bias. Additionally, the results are contingent upon the economic, political, social, and economic conditions prevalent within the country. Moreover, the study's scope is restricted by the relatively small number of interviews conducted, offering a representation of a portion of the industry but not an exhaustive account of the entire country's corporate landscape.

Another limitation of the study is the lack of statistical analysis beyond the qualitative assessment conducted. While the qualitative approach provided valuable insights into the status of OE practices in emerging countries and the factors influencing the maturity levels, a quantitative analysis could offer a more robust and generalizable understanding of the relationships between variables and the impact of different factors on OE. Future research could focus on incorporating quantitative methods such as statistical modeling, regression analysis, or correlation studies to provide a more in-depth and statistically sound evaluation of the factors influencing OE maturity levels in emerging markets.

A promising avenue for future research endeavors, aimed at bolstering the present study, involves conducting similar investigations in other emerging nations. By gauging the OE implementation status, the variable adoption timelines, and the perspectives of operational experts in different countries, an invaluable comparative analysis can be undertaken. Ultimately, the objective is to validate this research as a universally applicable OE roadmap proposal for emerging nations. Likewise, future research may delve into the costs entailed in transitioning from one tier to another and explore the economic impacts and other ramifications for companies aspiring to achieve champion OE status.

## Data availability statement

The data that has been used is confidential.

## CRediT authorship contribution statement

**Rafael Henriquez-Machado:** Writing – original draft, Methodology, Formal analysis, Conceptualization. **Andrés Muñoz-Villamizar:** Supervision. **Javier Santos:** Supervision.

## Declaration of competing interest

The authors declare that they have no known competing financial interests or personal relationships that could have appeared to influence the work reported in this paper.

## Figures and Tables

**Figure:**